# A new analysis of hypoxia tolerance in fishes using a database of critical oxygen level (*P*_crit_)

**DOI:** 10.1093/conphys/cow012

**Published:** 2016-04-27

**Authors:** Nicholas J Rogers, Mauricio A Urbina, Erin E Reardon, David J McKenzie, Rod W Wilson

**Affiliations:** af1 Biosciences, College of Life and Environmental Sciences, Geoffrey Pope Building, University of Exeter, Stocker Road, Exeter EX4 4QD, UK; af2 Centre for Marine Biodiversity Exploitation and Conservation (Marbec), UMR 9190 CNRS-Université Montpellier-Ifremer-IRD, Université Montpellier, Place Eugène Bataillon, Montpellier cedex 5 34095, France

**Keywords:** Carbon dioxide, critical oxygen tension, metabolic rate, oxygen and capacity limitation of thermal tolerance, physiological trait

## Abstract

Hypoxia is a common occurrence in aquatic habitats, and it is becoming an increasingly frequent and widespread environmental perturbation, primarily as the result of anthropogenic nutrient enrichment and climate change. An in-depth understanding of the hypoxia tolerance of fishes, and how this varies among individuals and species, is required to make accurate predictions of future ecological impacts and to provide better information for conservation and fisheries management. The critical oxygen level (*P*_crit_) has been widely used as a quantifiable trait of hypoxia tolerance. It is defined as the oxygen level below which the animal can no longer maintain a stable rate of oxygen uptake (oxyregulate) and uptake becomes dependent on ambient oxygen availability (the animal transitions to oxyconforming). A comprehensive database of *P*_crit_ values, comprising 331 measurements from 96 published studies, covering 151 fish species from 58 families, provides the most extensive and up-to-date analysis of hypoxia tolerance in teleosts. Methodologies for determining *P*_crit_ are critically examined to evaluate its usefulness as an indicator of hypoxia tolerance in fishes. Various abiotic and biotic factors that interact with hypoxia are analysed for their effect on *P*_crit_, including temperature, CO_2_, acidification, toxic metals and feeding. Salinity, temperature, body mass and routine metabolic rate were strongly correlated with *P*_crit_; 20% of variation in the *P*_crit_ data set was explained by these four variables. An important methodological issue not previously considered is the inconsistent increase in partial pressure of CO_2_ within a closed respirometer during the measurement of *P*_crit_. Modelling suggests that the final partial pressure of CO_2_ reached can vary from 650 to 3500 µatm depending on the ambient pH and salinity, with potentially major effects on blood acid–base balance and *P*_crit_ itself. This database will form part of a widely accessible repository of physiological trait data that will serve as a resource to facilitate future studies of fish ecology, conservation and management.

## Teaser

Data were compiled from the literature on the critical oxygen level (Pcrit), a hypoxia-tolerance trait, comprising 96 studies covering 151 fish species from 58 families. Salinity, temperature, body mass, and routine metabolic rate were highly correlated with Pcrit. The effects of temperature, CO2, acidification, metals and feeding are also assessed.

## Introduction

In recent decades, there has been growing concern regarding the increasingly widespread and frequent occurrence of hypoxia in aquatic environments, associated with the increased discovery of hypoxic zones globally (Diaz, 2001; Diaz and Breitburg, 2009; Zhang *et al*., 2010). Although periods of hypoxia can develop naturally in many aquatic systems, anthropogenic influences have been shown to be a major driver of hypoxic events in both freshwater and marine habitats (Friedrich *et al*., 2014). In particular, eutrophication associated with increased anthropogenic nutrient loading of lakes, rivers and coastal waters leads to blooms of algae and phytoplankton, the death of which subsequently fuels microbial respiration and the depletion of dissolved oxygen (Smith, 2003). Hypoxia has been shown to result in losses of biodiversity and to trigger widespread mortality events (Vaquer-Sunyer and Duarte, 2008). In the marine environment, more than 400 coastal systems have been reported as eutrophication-associated ‘dead zones’ (Diaz and Rosenberg, 2008). Global warming is likely to exacerbate hypoxia in aquatic systems owing to increased microbial respiration rates and reduced oxygen solubility with increasing water temperatures (McBryan *et al*., 2013). In addition, potential modifications to oceanic circulation linked to future climate change are predicted to result in greater stratification and ‘deoxygenation’ of the oceans (Keeling and Garcia, 2002; Keeling *et al*., 2009). In summary, in the future, reduced oxygen concentrations are predicted to occur more extensively, more frequently and for longer periods of time (IPCC, 2014). Fish are among the more hypoxia sensitive of aquatic taxa and, as such, the sequential loss of fauna from aquatic ecosystems during hypoxic events is commonly initiated by the loss or relocation of fish populations (Vaquer-Sunyer and Duarte, 2008). Understanding the physiological responses of individual organisms to environmental stressors, such as hypoxia, provides a mechanistic link between environmental change and population-level effects, which may be key to predicting future ecological impacts (Chown, 2012; Seebacher and Franklin, 2012; Cooke *et al*., 2013).

Fishes can show various behavioural responses to hypoxia, such as rising to the surface to breathe the uppermost layer of water in contact with air, increasing activity to escape the hypoxic area or decreasing activity to reduce oxygen demand (Chapman and McKenzie, 2009; Urbina *et al*., 2011; Domenici *et al*., 2012). Beyond these behavioural responses, fishes can engage numerous profound physiological responses, such as changes in ventilation, cardiac activity and haemoglobin–O_2_ binding (Richards, 2009). These physiological responses work primarily to sustain oxygen extraction from the environment in order to maintain aerobic ATP production. This allows the majority of fishes to maintain stable oxygen uptake rates across a wide range of ambient partial pressures of oxygen ($P_{O_{2}}$), a response known as ‘oxyregulation’ (reviewed by Perry *et al*., 2009). When, however, oxygen reduces to a threshold below which oxygen uptake rate cannot be maintained, oxygen uptake declines linearly with a decrease in ambient $P_{O_{2}}$, a response known as ‘oxyconforming’ (Pörtner and Grieshaber, 1993; Claireaux and Chabot, 2016). This threshold, when oxygen uptake transitions from regulation to conforming, is referred to as the critical $P_{O_{2}}$ (*P*_crit_; Beamish, 1964; Ultsch *et al*., 1978). As a measure of whole-animal oxygen extraction capacity, which varies extensively across species and among populations, *P*_crit_ is widely used to describe the degree of hypoxia tolerance in fishes (Ultsch *et al*., 1978; Chapman *et al*., 2002; Nilsson *et al*., 2007a,b; Mandic *et al*., 2009; reviewed by Chapman and McKenzie, 2009; Speers-Roesch *et al*., 2012).

**Figure 1:**
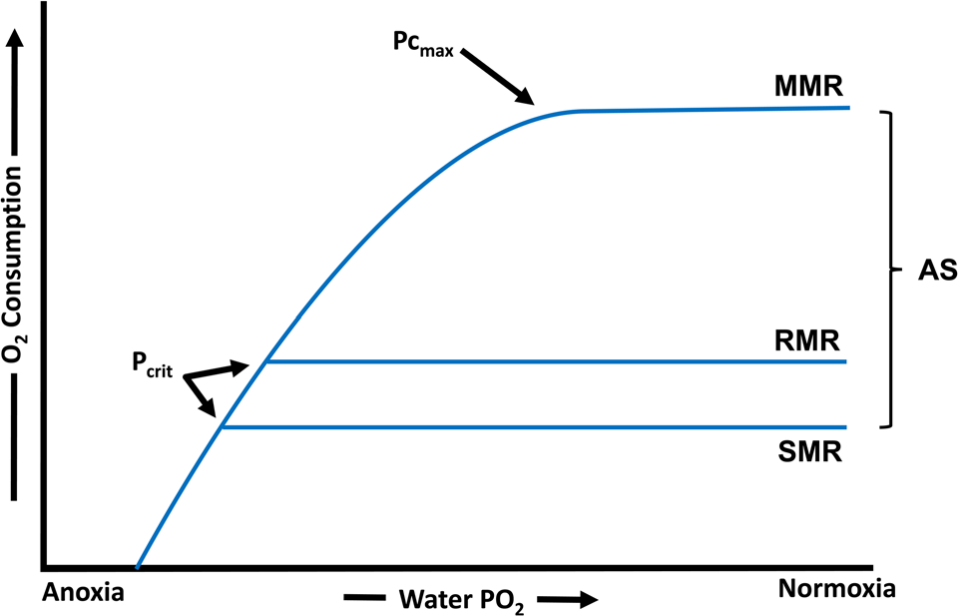
Diagram illustrating the conceptual idea of the effects of hypoxia on the standard metabolic rate (SMR), routine metabolic rate (RMR), maximal metabolic rate (MMR) and aerobic scope (AS) of an oxyregulator. This and may not apply to species with facultative metabolic depression below the critical oxygen level (*P*_crit_). *P*c_max_ is defined as the critical exeternal oxygen partial pressure at which oxygen supply no longer meets the maximum demand for oxygen (Portner, 2010).

Oxygen, the key variable in *P*_crit_ measurements, is used by aerobic organisms as an electron acceptor in order to drive the production of ATP. As such, the rate of oxygen uptake is widely considered as a proxy for the rate of aerobic metabolism, at least when in a steady state (Brown *et al*., 2004; Nelson, 2016). Standard metabolic rate (SMR) is the oxygen uptake rate of an entirely inactive, post-absorptive fish and reflects its minimal cost of living at a given temperature (Beamish and Mookherjii, 1964; Chabot *et al.*, 2016). Routine metabolic rate (RMR) provides a similar estimate of the cost of living but takes into account energy expended on maintaining posture and making the small movements that are typical of most fishes even when in a quiescent state (McBryan *et al*., 2013). In contrast, maximal metabolic rate (MMR) is the highest rate of oxygen uptake that can be attained in defined environmental conditions (Clark *et al*., 2013; Norin and Clark, 2016). The difference between SMR and MMR is referred to as aerobic scope and provides for the oxygen demands of higher functions, such as locomotion, growth, behaviour and reproduction (Farrell and Richards, 2009; Claireaux and Chabot, 2016). In the context of this aerobic hierarchy, several levels of critical $P_{O_{2}}$ are represented in Figure 1. As this conceptual diagram illustrates, MMR is the first rate to become limited as ambient oxygen decreases (*P*c_max_), from which point a decline in MMR leads to a reduction in aerobic scope. Secondly, the *P*_crit_ for RMR is reached, whereby oxygen supply cannot sustain even minimal levels of aerobic activity. Finally, the *P*_crit_ for SMR indicates that oxygen supply cannot meet even basic oxygen demands (Pörtner and Lannig, 2009; Claireaux and Chabot, 2016). Below this threshold, anaerobiosis or suppression of metabolic rate are required to sustain life (Richards, 2009). Each of the three levels of *P*_crit_ may indicate the difference between mortality and survival. If so, *P*_crit_ may have major implications for the fitness of fishes living in environments prone to hypoxia and, as such, each of these levels can be considered as functional traits (McGill *et al*., 2006; Claireaux and Chabot, 2016).

The examination of trait variation across populations and communities, and its ecological implications, are increasingly becoming the basis for predicting and potentially mitigating the effects on biodiversity of environmental change (Chown, 2012). Such trait-based approaches are facilitated by the collection and dissemination of trait data. Large-scale multi-trait databases have been compiled for various taxa, including plants (Kattge *et al*., 2011), mammals (Jones *et al*., 2009), marine polychaetes (Faulwetter *et al*., 2014) and North American freshwater fishes (Frimpong and Angermeier, 2009). As a quantifiable measure of hypoxia tolerance that is measured on individuals and is applicable at population level, *P*_crit_ is useful for incorporation into trait-based approaches to the conservation physiology of fishes (Frimpong and Angermeier, 2009).

The field of fish physiology has generated a large body of literature on *P*_crit_, across a wide range of species and in highly variable abiotic and biotic conditions (Perry *et al*., 2009). Owing to the discrete and nuanced nature of each study, it is challenging to make broad generalizations. The aims of the present work were as follows: (i) to assemble a database of the *P*_crit_ values reported for fishes, from published literature, in a format suitable for future incorporation into multi-trait-based analyses; (ii) to analyse the data to identify how biotic and abiotic factors (particularly temperature) interact with hypoxia and affect *P*_crit_; and (iii) to appraise methodologies for measuring *P*_crit_ critically, and thereby evaluate its usefulness for quantifying hypoxia tolerance in fishes. This new analysis not only provides an opportunity for further quantitative considerations but also serves as a tangible link between the physiology and the conservation of fishes.

## Methods

### Literature search

The citation and abstract indexes, Scopus^®^ and Web of Science^®^, were used to collect relevant peer-reviewed literature. The literature search was conducted in December 2014 using the following terms: ‘critical oxygen’, ‘critical *P*O_2_’, ‘oxygen threshold’, ‘*P*_crit_’, ‘oxyregulate’, ‘oxyconform’ or ‘hypoxia tolerance’. Approximately 400 papers from relevant subject areas were identified. Each of these articles was individually assessed for relevance based on their title and abstract. Finally, 144 papers were downloaded for a full read of the manuscript. Of these, only 96 papers reported *P*_crit_ measurements in at least one fish species.

### Database construction

In order to maximize the future usefulness of the database and to ensure that it fully reflects the variation in abiotic/biotic conditions in which *P*_crit_ has previously been measured in fishes, it was necessary to extract multiple parameters from each study. For each *P*_crit_ entry, 66 columns summarize information on the species and origin, acclimation parameters, animal characteristics, experimental method, results, statistical analyses, general comments and bibliographic information (Table 1). The database was constructed as a single Microsoft Excel file, with individual columns for each parameter and rows for each *P*_crit_ determination in a particular species or treatment group. As such, a single study may occupy several rows depending on the number of treatment groups and/or species for which *P*_crit_ is reported. Values for *P*_crit_ were reported in a variety of different oxygen units across the literature (millimetres of mercury, torr, percentage air saturation, milligrams of oxygen per litre and micromolar), but were converted here to a partial pressure of oxygen (in kilopascals) based on oxygen solubility values reported by Green and Carrit (1967) and assuming standard atmospheric pressure at sea level (760 mmHg), if not otherwise reported. Likewise, all values of oxygen uptake rate were converted to milligrams of oxygen per kilogram per hour. To enable unbiased inter-species comparison, a subset of the full database was produced, which included only those *P*_crit_ measurements made in fishes meeting the following conditions: (i) in an unfed or post-absorptive state; (ii) undergoing no additional (to hypoxia) abiotic stressor; and (iii) where temperature acclimation lasted for >2 days.

**Table 1:** List of the parameters incorporated into the database alongside each reported critical oxygen level value

Species and origin	Stock acclimation	Sample characteristics	Experimental method	Results	Statistical analysis	Comments and reference
Family	Holding time	Sample size	Respirometry type	Oxy regulating or conforming	Statistical method	Comments
Genus	Acclimation temperature	Mean mass	BMR/RMR/SMR/MMR	$M_{O_{2}}$	*P*_crit_ calculation method	Reference
Species	Acclimation salinity	Mass SD	Determination method	Critical $P_{O_{2}}$	SMR determination	Year
Origin	$P_{O_{2}}$ units	Mass SEM	Swimming speed	Critical $P_{O_{2}}$ range		Corresponding Author
Latitude and longitude	Acclimation $P_{O_{2}}$	Mass range upper	Hypoxia method	Critical $P_{O_{2}}$ SD		DOI
	Acclimation pH	Mass range lower	Rate of hypoxia onset	Critical $P_{O_{2}}$ SEM		Full citation
	Acclimation time	Mean length	$P_{O_{2}}$ set-point time	Critical $P_{O_{2}}$ units		
	Diet	Length SD	Minimal $P_{O_{2}}$	Air breathing threshold		
	Energy content	Length SEM	$P_{O_{2}}$ unit	Common $P_{O_{2}}$ units		
	Ration unit	Length range upper	Salinity			
	Ration size	Length range lower	Temperature			
	Photoperiod (light:dark)	Life stage	pH			
	Feeding regimen	Sex	$P_{CO_{2}}$			
		Last feed	Photoperiod (light:dark)			
			Access to air			

Abbreviations: BMR, basal metabolic rate; DOI, digital object identifier; MMR, maximal metabolic rate; $M_{O_{2}}$, oxygen uptake rate; $P_{CO_{2}}$, partial pressure of carbon dioxide; *P*_crit_, critical oxygen level; $P_{O_{2}}$, partial pressure of oxygen; RMR, routine metabolic rate; SMR, standard metabolic rate.

### Database analysis

The frequency of *P*_crit_ measurements across families and climate zones was calculated based on the full database. However, comparisons of *P*_crit_ values were made using the subset ‘control’ database described above. Based on the latitude of where the studies were conducted, each entry was labelled as tropical, sub-tropical, temperature or polar. Analysis of variance was used to test for an effect of climate zone on *P*_crit_ using the Sidak *post hoc* test.

Potential influences of varying respirometry methodologies and hypoxia exposure methods on *P*_crit_ were explored using the subset ‘control’ database, in which there are 297 data points. Similar to the full database, the majority of studies measured *P*_crit_ using closed static respirometry on individual fish, where oxygen is reduced via the oxygen consumption of the fish (*n* = 202). Where there were sufficient data to compare methods between respirometry methods within a species, a Student’s unpaired *t*-test was used to compare between groups. It was not possible to test for differences in hypoxia exposure methods within species because there were insufficient data from at least two methods.

Stepwise multiple linear regression analysis was used to develop a model for predicting *P*_crit_ based on biotic (body mass, RMR) and abiotic (temperature, salinity) variables. Earlier analysis detected no significant within-species effect of respirometry method (closed or flow through) on *P*_crit_, and it was therefore not included in the linear regression model. Acclimation variables such as temperature, $P_{O_{2}}$ and salinity were not included in this analysis because they were very highly correlated with the equivalent variables reported during the trials. Minimal $P_{O_{2}}$ was not included in the model because it is driven by *P*_crit_.

As the multivariate model identified salinity as a relevant factor, the potential effect of salinity on *P*_crit_ was explored further by comparing *P*_crit_ values measured in seawater (150 entries from 82 species) with *P*_crit_ values measured in freshwater (116 entries from 50 species). This approach was taken because most of the studies were conducted either in freshwater [∼0.1 practical salinity units (PSU)] or seawater (∼30–38 PSU). Values of *P*_crit_ were calculated as the partial pressure of oxygen (in kilopascals) and as the concentration of oxygen (in milligrams per litre), using the solubility coefficient based on experimental temperature and salinity (Green and Carrit, 1967). Potential differences between groups were then tested by a Mann–Whitney *U*-test, because normality assumptions were violated.

## Results and discussion

### Database coverage

Of the 96 studies reviewed, 331 measurements of *P*_crit_ across 151 species were incorporated into the database. Across the global database, 58 families are represented, with Cyprinidae (*n* = 44), Pomacentridae (*n* = 41), Gobiidae (*n* = 24), Cichildae (*n* = 23), Salmonidae (*n* = 19), Cottidae (*n* = 18), Apogonidae (*n* = 17), Percidae (*n* = 13) and Sparidae (*n* = 12) the most frequently represented. Freshwater and marine (including euryhaline) species account for 40 and 60% of *P*_crit_ entries, respectively. Water temperatures at which *P*_crit_ values were determined ranged between −1.5 and 36°C, with a mean (±SD) of 21.7 ± 7.6°C. Values for *P*_crit_ over the entire data set ranged between 1.02 kPa (*Pseudocrenilabrus multicolor victoriae*; Reardon and Chapman, 2010) and 16.2 kPa (*Solea solea* larvae; McKenzie *et al.*, 2008) with a mean (±SD) *P*_crit_ in the ‘control’ data set of 5.15 ± 2.21 kPa. Plots of species and their reported *P*_crit_ values from the subset data set are provided in the Supplementary Data (Supplementary Fig. 1).

The geographical coverage of the database includes at least one entry from every continent, although North America, Europe and Australasia are by far the most heavily represented and, when combined, account for 87% of *P*_crit_ entries. Perhaps unsurprisingly, most studies of *P*_crit_ in fishes have been concentrated around the major fish physiology research groups in Europe, North America and Australia. Arguably, this introduces an element of bias into the database, given the incomplete representation of all habitats and species at a global scale. Based on the full database, tropical studies are the most frequently represented (*n* = 125 *P*_crit_ measurements, dominated by Lizard Island Research Station, Australia, *n* = 98), followed by subtropical (*n* = 104) and temperate regions (*n* = 100), dominated by Canada and Europe. The polar regions are the most under-represented (*n* = 2). Within the subset ‘control’ database, there was a significant difference in mean *P*_crit_ across climatic regions (ANOVA, *F*_2,297_ = 4.054, *P* = 0.018), where tropical fishes had the lowest *P*_crit_ (mean ± SEM: 4.92 ± 0.190 kPa) < sub-tropical fishes (5.0 ± 0.24 kPa) < temperate fishes (5.74 ± 0.24 kPa). However, the Sidak *post hoc* test suggested that *P*_crit_ values for tropical fishes were significantly lower only than temperate fishes (*P* = 0.021). There was no difference in mean *P*_crit_ between subtropical and either tropical (*P* = 0.991) or temperate *P*_crit_ (*P* = 0.085). Owing to low sample size, the polar *P*_crit_ values were not included in the ANOVA across temperatures but, interestingly, had a higher mean *P*_crit_ than the other three climatic zones (7.9 ± 1.6 kPa).

Additionally, the species studied tend to be those conducive to respirometry trials. In particular, large, active or highly sensitive species, such as those of the Scombridae family (tuna, mackerels and bonitos) are generally under-represented in the literature (Blank *et al*., 2007). For example, the majority of *P*_crit_ values reported in the database were measured on fish <1 kg body mass.

### Methodology used to determine critical oxygen level

The relationship between ambient $P_{O_{2}}$ and oxygen uptake in fishes has been investigated since the study of Keys (1930). Even at that early stage, there was considerable discussion among physiologists regarding the validity of different methodologies. Technological developments, particularly methods for measuring dissolved oxygen content such as galvanic oxygen electrodes and, more recently, fibre-optic sensors, have made the performance of high-resolution measurements of oxygen uptake in fishes increasingly common (Clark *et al*., 2013; Nelson, 2016). Nevertheless, the literature examined for the purpose of building this database is characterized by considerable variation in terms of methods used to determine *P*_crit_. For example, the majority of studies (56%) used closed respirometry for *P*_crit_ estimates, 21% used flow-through respirometry, 20% used intermittent respirometry, and 3% used other approaches, such as indirect estimation of gill oxygen uptake (Table 2). Most studies (70%) depleted ambient oxygen through the fish’s own respiration, whereas 30% of studies bubbled nitrogen gas into the water to reduce ambient oxygen levels. The majority of studies (80%) measured RMR for *P*_crit_ estimates; the remaining 20% measured SMR. These methodological differences and their implications are important to consider when interpreting collated *P*_crit_ data.

**Table 2:** The breakdown of the number of data points representing each respirometry type and oxygen removal method in the subset database

	Oxygen depletion method					
Respirometry type	Fish respiration	N_2_ equilibration	N_2_ and O_2_ equilibration	N_2_ and CO_2_ equilibration	N_2_, O_2_ and air equilibration	Total

Closed static (individual)	202	1	0	0	0	203
Closed static (grouped)	13	0	0	0	0	13
Closed flow-through (individual)	13	14	0	0	3	30
Intermittent flow (individual)	13	26	2	1	0	42
Mesocosm (grouped, large tuna)	0	1	0	0	0	1
Open flow-through (grouped)	7	0	0	0	0	7
Opercular mask	1	0	0	0	0	1

Closed respirometry, whereby the fish is placed within a sealed chamber from which water is intermittently sampled for measurement of dissolved oxygen content, provides the simplest method of measuring oxygen uptake rate (Steffensen, 1989), as follows:

$$M_{O_{2}}=[(V_{r}-V_{f})\times \Delta O_{2}]÷(\Delta t\times \mathrm{bw}),$$

where $M_{O_{2}}$ represents oxygen uptake rate, *V*_r_ is respirometer volume, *V*_f_ is fish volume, ΔO_2_ is change in ambient oxygen content, *t* is time, and bw is fish mass (‘body weight’). Importantly, water needs to be recirculated within the chamber to ensure adequate mixing, thus preventing the stratification of dissolved oxygen within the chamber (Keys, 1930). Whether spontaneous movements and ventilation are sufficient to provide mixing depends on the species and achieving the correct fish-to-respirometer volume ratio. For closed determinations of *P*_crit_, hypoxia is generated by allowing the fish to deplete available oxygen through its own respiration, therefore negating the need to strip dissolved oxygen from the water artificially through equilibration with nitrogen. For this reason, closed respirometry is particularly useful for conducting measurements of *P*_crit_ in the field or at remote locations where facilities such as a supply of N_2_ may not be readily available (Rosenberger and Chapman, 2000; Nilsson *et al*., 2007b).

However, there are several important considerations regarding the use of closed respirometry for determination of *P*_crit_. For instance, the rate of oxygen depletion during closed respirometry is determined by the ratio of fish size (or oxygen uptake rate) to respirometer volume. A lack of control over the development of hypoxia can be problematic in comparative studies that use the same respirometer to measure *P*_crit_ in fish of different size and/or metabolic rate. As an illustrative example, the depletion of oxygen levels from 20 to 1 kPa by Australian barramundi (*Lates calcarifer*) took between 1.5 and 4 h depending on the temperature (26 or 36°C; Collins *et al*., 2013). From our database, it is evident that there is very little, if any, standardization in terms of the rate of oxygen depletion between *P*_crit_ studies, irrespective of which respirometry method is employed. This is in contrast to measurements of other physiological threshold traits, such as the determination of critical temperature, which tends to be made at consistent warming or cooling rates among studies (0.2–0.3°C min^−1^; Beitinger *et al*., 2000; Mora and Maya, 2006; Murchie *et al*., 2011). It is unclear whether the rate of decline in ambient oxygen will significantly affect *P*_crit_, but it is likely that a longer time scale would allow for greater respiratory adjustments, and hence, reveal lower *P*_crit_ values than more acute hypoxic exposures. Indeed, our own anecdotal observations in European flounder (*Platichthys flesus*) suggest that these fish tend to oxyconform across the entire range of ambient $P_{O_{2}}$ when exposed to a very rapid reduction of oxygen (from 21 to 2 kPa in <2 h).

A further issue associated with closed respirometry is the build-up of the waste products of metabolism, in particular CO_2_ (Keys, 1930; Steffensen 1989; Urbina *et al*., 2012). It has been argued that the level of CO_2_ accumulation within a closed respirometer is unlikely to impact on CO_2_ excretion by fishes significantly, given that they normally exhibit a blood partial pressure of CO_2_ ($P_{CO_{2}}$) of around 2–4 mmHg, much higher than normal ambient levels (Ishimatsu *et al*., 2005; Nilsson *et al*., 2007a). However, a precedent has been set, albeit at more severe levels of hypercarbia (2.25–20 mmHg), to show that elevated $P_{CO_{2}}$ can increase *P*_crit_ in European eels (*Anguilla anguilla*; Cruz-Neto and Steffensen, 1997), although no effect on *P*_crit_ was observed when eels were given enough time to acclimate fully in terms of acid–base regulation (McKenzie *et al*., 2003), or in spot fish (*Leiostomus xanthurus*) and mummichog (*Fundulus heteroclitus*; Cochran and Burnett, 1996). Given the potential influence of hypercarbia, it would be prudent to report any change in water $P_{CO_{2}}$ alongside values for *P*_crit_ that have been determined through closed respirometry, but this has rarely been the case throughout the existing literature. A single study so far has evaluated this potential confounding factor in determining *P*_crit_, but in this unusual oxyconforming species (inanga, *Galaxias maculatus*) elevated $P_{CO_{2}}$ had no effect on oxygen uptake rate at any level of ambient oxygen (Urbina *et al*., 2012). Furthermore, the authors pointed out that the effect of CO_2_ on $M_{O_{2}}$ in fishes appears to be species specific (Gilmour, 2001; Ishimatsu *et al*., 2008).

**Figure 2:**
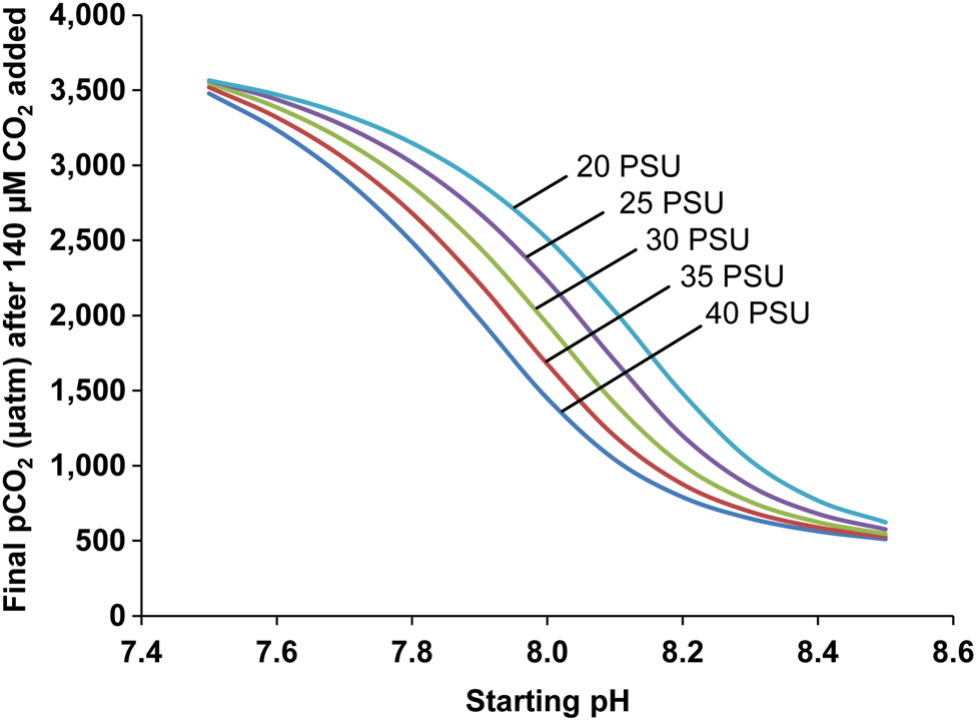
Model of the estimated partial pressure of carbon dioxide ($P_{CO_{2}}$) reached, in water of different salinities and starting pH values, after the addition of 140 µM CO_2_. The value of 140 µM approximates the increase in total CO_2_ attributable to excretion by a fish at 15°C during a closed respirometry experiment. In this theoretical example, the oxygen level is allowed to decline as a result of respiration from a normoxic partial pressure of >20 kPa (∼245 µM) to a common *P*_crit_ value of ∼6 kPa (∼74 µM), and we have assumed a respiratory quotient (CO_2_ excreted ÷ O_2_ consumed) of 0.85 for fish (Kieffer *et al*., 1998). At each starting pH, the total alkalinity (TA) and total CO_2_ were calculated from the pH and assuming equilibration with atmospheric $P_{CO_{2}}$ (395 µatm). When excreted CO_2_ is dissolved in water, the total CO_2_ increases accordingly (in this case, by 140 µM) but TA remains unchanged (Riebesell *et al*., 2010). For each starting pH, we therefore used the CO2sys program (for the national bureau of standards pH scale) to calculate the final $P_{CO_{2}}$ that would result from increasing total CO_2_ by 140 µM while TA remained constant. This was repeated for salinities of 20, 25, 30, 35 and 40 practical salinity units (PSU) and starting pH values of 7.5–8.5 to cover ranges experienced in many marine laboratories.

An important issue that does not appear to have been considered previously is that the extent to which $P_{CO_{2}}$ increases within a closed respirometer will be highly dependent on the starting water chemistry, in particular pH and salinity (Fig. 2). A higher seawater pH indicates a greater total alkalinity (TA). In turn, this gives increased capacity for buffering added CO_2_ and limiting the increase in $P_{CO_{2}}$ for a given increase in total CO_2_ attributable to net excretion by the fish in a respirometer. Therefore, the lower the starting water pH, the larger the overall change in $P_{CO_{2}}$ over the course of the *P*_crit_ measurement. From the models shown in Figure 2, it is clear that pH has a massive influence on the ambient $P_{CO_{2}}$ reached within such a closed respirometry scenario, with final $P_{CO_{2}}$ values ranging by 5-fold, from ∼650 µatm (0.49 mmHg) to ∼3500 µatm (2.66 mmHg) at the highest (8.5) and lowest (7.5) starting pH values shown, respectively. Note that even the lowest of these final $P_{CO_{2}}$ values has been shown (in experiments designed to mimic future ‘ocean acidification’ scenarios) to have significant detrimental effects in fishes (Munday *et al*., 2009). When the starting pH is low, the highest $P_{CO_{2}}$ values of ∼3500 µatm occur, which are more than 3.5 times higher than the ‘business as usual’ for end-of-century global CO_2_ projections (representative concentration pathway scenario 8.5; Meinshausen *et al*., 2011). It is also relevant to note that salinity has a major modulating effect, in particular within the middle of the range of starting pH values. For example, at a starting pH of 8.0, the final $P_{CO_{2}}$ will vary from slightly <1500 µatm (1.14 mmHg) at the highest salinity (40 PSU) to >2500 µatm (1.90 mmHg) at the lowest salinity (20 PSU).

The larger ambient $P_{CO_{2}}$ values indicated above would certainly be expected to cause significant blood acid–base disturbance during the time scale of a typical closed respirometry experiment (minutes to hours) and thus have the potential to influence *P*_crit_ via alterations in the oxygen binding affinity of haemoglobin. It is therefore important to recognize this variability in $P_{CO_{2}}$ when conducting closed respirometry experiments to determine hypoxia tolerance, and particularly, when interpreting *P*_crit_ measurements.

Flow-through respirometry is a technique whereby oxygen content of the inflowing (O_2_,in) and outflowing (O_2_,out) water is continuously measured at a fixed water flow rate through the respirometer (*F*_w_). By application of the Fick principle, oxygen uptake ($M_{O_{2}}$) is determined by:

$$M_{O_{2}}=F_{w}(O_{2},in-O_{2},\mathrm{out})÷bw.$$

Although flow-through respirometry avoids the accumulation of metabolites in the chamber, it suffers from problems primarily related to the ‘wash-out’ effect, whereby a significant lag can develop between changes in the fish’s real $M_{O_{2}}$ and changes in observed O_2_,out. The degree of wash-out depends on the dilution factor, which is a function of water mixing, volume and flow rate (Steffensen, 1989).

Intermittent flow-through respirometry is generally considered the ideal method of $M_{O_{2}}$ determination in fishes because it involves none of the problems associated with closed or flow-through techniques (Steffensen, 1989; Clark *et al*., 2013). The term ‘intermittent’ or ‘semi-closed’ in this context refers to the transitioning between a closed phase for determination of $M_{O_{2}}$ and a flush phase for restoring O_2_ to a set level and removing metabolites from the respirometer. As the equipment and software for automating flush–recirculation cycles and simultaneous data acquisition from multiple chambers have become more sophisticated and widely available, intermittent flow-through respirometry has been increasingly used (Svendsen *et al.*, 2016). However, *P*_crit_ measurements via this preferred technique account for only 20% of values incorporated into the present database.

Flow-through techniques allow for the supply of hypoxic water to the respirometry chamber. This hypoxic water can be produced by bubbling with N_2_ via a solenoid valve linked to an O_2_ probe (Schurmann and Steffensen, 1997) or by bubbling with set gas mixtures of variable O_2_ and N_2_ content. Both methods allow for finer control of the hypoxic exposure compared with allowing the fish to deplete ambient oxygen levels dependent on its own $M_{O_{2}}$. Progressive hypoxia can be generated in a stepwise fashion such that multiple $M_{O_{2}}$ measurements can be made at a specific $P_{O_{2}}$, thereby increasing the likelihood of determining an $M_{O_{2}}$ that is representative of true SMR or RMR (Rantin *et al*., 1993)_._

Using the present database, we were able to explore differences in respirometry methods within three species, Atlantic salmon (*Salmo salar*), common carp (*Cyprinus carpio*) and Nile tilapia (*Oreochromis niloticus*), for which the sample size for at least two methods was greater than *n* > 2. Between closed static or closed flow-through respirometers, there was no difference in *P*_crit_ of common carp (Student’s unpaired *t*-test, *t* = 1.429, d.f. = 6, *P* = 0.203). Likewise, between closed, static respirometers (individual fish) and open flow respirometry (with grouped fish), there was no difference in *P*_crit_ in Atlantic salmon (Student’s unpaired *t*-test, *t* = −0.678, d.f. = 8, *P* = 0.517). There was no difference in *P*_crit_ between closed, flow-through or intermittent flow-through respirometry within Nile tilapia (Student’s unpaired *t*-test, *t* = −0.644, d.f. = 6, *P* = 0.543). In both Atlantic salmon and common carp, oxygen levels were reduced by the respiration of the fish, whereas in Nile tilapia the oxygen was reduced by nitrogen equilibration. A direct comparison in the shiner perch (*Cymatogaster aggregata*) found, however, that *P*_crit_ measured by intermittent flow-through respirometry was significantly lower than that measured by closed respirometry (Snyder *et al.*, 2016). Thus, more direct comparisons are needed to investigate whether the two most common methodologies might provide different estimates of *P*_crit_.

To determine *P*_crit_, $M_{O_{2}}$ is plotted against ambient $P_{O_{2}}$ in order to identify the inflection point at which $M_{O_{2}}$ transitions from being independent of ambient oxygen to dependent on ambient oxygen. Within this procedure, a great deal of subtle variation exists among studies. Most obvious is the differential use of SMR or RMR, with the majority (84%) of studies reporting a *P*_crit_ for RMR. Arguably, the *P*_crit_ exhibited for RMR is more ecologically relevant, given that this level of $M_{O_{2}}$ is likely to be exhibited most of the time in the field (Ultsch *et al*., 1978; Pörtner, 2010). Indeed, for some highly active species, such as salmonids, *P*_crit_ determined during active swimming may be most useful in considering the ecological implications of hypoxia (Fry, 1957). Activity level may affect *P*_crit_ in unexpected ways, such as in the Adriatic sturgeon (*Acipenser naccarii*), which exhibits a well-developed ability to oxyregulate (*P*_crit_ = 4.9 ± 0.5 kPa) when permitted to swim at a low sustained speed but oxyconforms across the entire range of declining ambient oxygen when its activity is restricted in a static respirometer (McKenzie *et al*., 2007). Some species exhibit a relatively high *P*_crit_ for RMR at a $P_{O_{2}}$ that is well above the *P*_50_ (half of the hemoglobin oxygen binding sites are saturated with oxygen) of their haemoglobin. In these instances, *P*_crit_ may indicate a behavioural change and not simply a physical limitation of oxygen supply (McBryan *et al*., 2013).

Of the studies that determine the *P*_crit_ for SMR, the methods used for quantifying SMR vary considerably. Some studies use the single lowest $M_{O_{2}}$ value recorded at normoxia, whereas others take the average of a set number of the lowest $M_{O_{2}}$ values (Iversen et al., 2010). More sophisticated and robust methods involve extrapolating the average $M_{O_{2}}$ measured at specified swimming speeds back to zero activity (Wilson *et al*., 1994; Cook *et al*., 2014) or the use of percentiles and frequency distributions to assess all normoxic $M_{O_{2}}$ data (Dupont-Prinet *et al*., 2013). As the critical level for basal metabolism, *P*_crit_ determinations based on SMR should theoretically reflect a true physiological limitation of oxygen extraction capacity (McBryan *et al*., 2013), although this may not be true in species for which metabolic depression below *P*_crit_ has a facultative component. Given that the *P*_crit_ for RMR is likely to be encountered at higher $P_{O_{2}}$ than that for SMR (Fig. 1), intra- or inter-species comparisons among studies reporting different levels of *P*_crit_ may not be entirely valid. Whether SMR or RMR measurements are used to reflect normoxic $M_{O_{2}}$, it is essential that sufficient time is allowed for the fish to acclimate to the respirometry chamber; otherwise, apparent reductions in $M_{O_{2}}$ as hypoxia develops may be an artefact of increasing habituation rather than true oxyconforming (Nilsson *et al*., 2004).

The method used to establish the point of intersection between continuous oxyregulation and oxyconforming $M_{O_{2}}$ data is also inconsistent among studies. The slope of these lines will determine the *P*_crit_ and vice versa; therefore, determining which data points should be included within each line is critical to establishing an accurate estimate of *P*_crit_ (Yeager and Ultsch, 1989). This can be achieved graphically by fitting a least-squares linear regression through data points that show a progressive decline in $M_{O_{2}}$ such that it intersects with a regression line fitted through normoxic $M_{O_{2}}$ data (Monteiro *et al*., 2013). A number of mathematical methods for performing so-called piece-wise or segmented linear regression analyses are available, which provide greater robustness to estimates of *P*_crit_ and are used in the majority of studies incorporated into the present database (Nickerson *et al*., 1989; Yeager and Ultsch, 1989; Leiva *et al*., 2015). These approaches assume that the response of $M_{O_{2}}$ to declining $P_{O_{2}}$ is biphasic and consists of two entirely linear elements, with an abrupt transition between the two. Such assumptions are not necessarily met by real-world data, and indeed, concentration-dependent reaction kinetics make truly linear relationships between $M_{O_{2}}$ and $P_{O_{2}}$ unlikely (Marshall *et al*., 2013). Recent developments in non-linear regression techniques are now being promoted as a more accurate approach to determining biological thresholds such as *P*_crit_ (Stinchcombe and Kirkpatrick, 2012; Marshall *et al*., 2013).

### Critical oxygen level as a hypoxia tolerance trait

A low *P*_crit_ is generally associated with greater hypoxia tolerance because it indicates a higher capacity for oxygen extraction and tissue delivery at low $P_{O_{2}}$ (Mandic *et al*., 2009). Maintaining aerobic metabolism during hypoxia is advantageous because it is up to 30-fold more efficient than anaerobic ATP production (per unit substrate consumed) and avoids accumulation of the deleterious by-products (e.g. H^+^) of anaerobic metabolism (Richards, 2009). Hypoxia-induced physiological modifications that increase oxygen extraction capacity, such as increased gill surface area (Nilsson, 2007) and haemoglobin–O_2_ binding (Brix *et al*., 1999), are observed in fishes that frequently encounter hypoxia, suggesting that maintaining aerobic metabolism is a primary hypoxia survival strategy (Mandic *et al*., 2009). However, when ambient $P_{O_{2}}$ declines below *P*_crit_, survival depends on the availability of substrate for O_2_-independent ATP production (primarily glycolysis) and the ability to reduce metabolic demand (Richards, 2009).

How long a fish can maintain a balance between ATP demand and supply below its *P*_crit_, and thus delay the onset of cellular dysfunction, necrosis and subsequent death, is a key component of hypoxia tolerance (Nilsson and Östlund-Nilsson, 2008; Urbina and Glover, 2012; Speers-Roesch *et al*., 2013). Speers-Roesch *et al*. (2013) showed that *P*_crit_ does not entirely predict hypoxia tolerance at lower oxygen levels. The authors used three species of sculpin (*Blepsias cirrhosis*, *Leptocottus armatushave* and *Oligocottus maculosus*), which exhibit different *P*_crit_ values (1.76, 1.48 and 1.03 kPa, respectively), and exposed them to hypoxia levels that were 30% below each of their respective *P*_crit_ values while recording the time to loss of equilibrium. The loss of equilibrium was consistent between only two of the three species (*L. armatushave* and *O. maculosus*). Similar relative hypoxia exposures in the epaulette shark (*Hemiscyllium ocellatum*) and shovelnose ray (*Aptychotrema rostrata*) revealed lower lactate accumulation in epaulette sharks, indicating enhanced metabolic depression in this species (Speers-Roesch *et al*., 2012). Furthermore, Nilsson and Östlund-Nilsson (2008) showed that *P*_crit_ did not correlate with body mass in juvenile and adult damselfish (Pomacentridae) ranging between 10 mg and 40 g but that smaller fish were much less tolerant to hypoxia below *P*_crit_, owing to their limited capacity for meeting ATP demand through anaerobic metabolism. These findings were further supported in *G. maculatus* (Urbina and Glover, 2013). These results illustrate the benefit of considering *P*_crit_ alongside other methods of determining hypoxia tolerance, such as measurements of tissue-specific lactate accumulation and determinations of the loss of equilibrium of 50% of the fish, in order to assess overall hypoxia tolerance (Urbina and Glover, 2013; Speers-Roesch *et al*., 2013; Claireaux and Chabot, 2016).

A recent review by Salin *et al.* (2015) argues that whole-animal oxygen consumption measurements may provide only a partial proxy for energy metabolism because of variation, within and between individuals, in the amount of ATP produced per molecule of oxygen consumed by mitochondria (P/O ratio). Environmental factors such as ambient temperature, food intake and diet composition have been shown both to increase and to decrease P/O ratios in the mitochondria of a variety of organisms (Salin *et al.*, 2015). Hence, conclusions based on oxygen consumption rate alone could lead to misleading conclusions regarding respiratory performance during environmental changes. To our knowledge, the effect of hypoxia on P/O ratios in fish has yet to be investigated, and as such, provides an interesting avenue for further research.

As a hypoxia-tolerance trait, low *P*_crit_ can often, but not always, indicate an ability to survive in hypoxic water. It does not consider the use of hypoxia-avoidance strategies, such as adaptations for emersion, aquatic surface respiration and air breathing (Chapman and McKenzie, 2009). The inanga (*G. maculatus*), which inhabits lowland streams prone to severe hypoxia, is a rare example of a fish species that appears to be an entirely obligate oxyconformer and thus demonstrates no discernible *P*_crit_ (Urbina *et al*., 2012). Likewise, several species of Gymnotiform electric fishes from South America, which inhabit naturally hypoxic floodplain pools, also appear to be obligate oxyconformers with no *P*_crit_ (Reardon E. E., personal communication), an observation that is also anecdotally supported in *Brachyhypopmus brevirostris* (Crampton, 1998). In some of these species, such as the inanga, a lack of scales and a large surface area-to-volume ratio indicate a high capacity for cutaneous O_2_ uptake whilst emersed, and hence, provide a short-term means to escape aquatic hypoxia (Urbina *et al*., 2011). The oxygen thresholds for aquatic surface respiration, air breathing and emergence were incorporated into the database, but only where they have been reported alongside *P*_crit_ measurements. Such examples demonstrate the limitation of *P*_crit_ as a universal and comparative measure of hypoxia tolerance between species and emphasize the benefit of multi-trait-based approaches.

### Biotic and abiotic interactions

Environmental stressors, such as hypoxia, rarely occur in isolation, and the interaction between stressors is of key concern in the context of predicting the ecological impacts of future environmental change (Crain *et al*., 2008). As a typical threshold effect, the response of fish to hypoxia is likely to result in ‘ecological surprises’, whereby seemingly resilient populations suddenly collapse once a critical threshold is crossed (McBryan *et al*., 2013). Additive or synergistic interactions with hypoxia could hasten the arrival of such thresholds, meaning that small environmental shifts could result in large effects on the performance of a population. Theoretically, any abiotic or biotic factor that affects either oxygen supply (cardiorespiratory capacity) or oxygen demand (metabolic rate) of an individual, and the balance therein, will have implications for its hypoxia tolerance. As an indicator of hypoxia tolerance, the effects of a wide range of abiotic and biotic interactions on *P*_crit_ in fish have been published (Table 3).

**Table 3:** Summary of biotic and abiotic factors and their interactions with the intra-species critical oxygen level as reported by studies included in the database

Variable	Species	Effect on *P*_crit_	Reference
Increasing temperature			
	*Gadus morhua*	Increase	Schurmann and Steffensen (1997)
	*Lates calcarifer*	Increase	Collins *et al*. (2013)
	*Scyliorhinus canicula*	Increase	Butler and Taylor (1975)
	*Salmo salar*	Increase	Barnes *et al*. (2011)
	*S. salar*	Increase	Remen *et al*. (2013)
	*Dentex dentex*	Increase	Cerezo Valverde et al. (2006)
	*Tautogolabrus adspersus*	Increase	Corkum and Gamperl (2009)
	*Gadus ogac*	Increase	Corkum and Gamperl (2009)
	*Bellapiscis medius*	Increase	Hilton *et al*. (2008)
	*Bellapiscis lesleyae*	Increase	Hilton *et al*. (2008)
	*Morone saxatilis*	Increase	Lapointe *et al*. (2014)
	*Carassius carassius*	Increase	Sollid *et al*. (2005)
	*Gobiodon histrio*	Increase	Sørensen et al. (2014)
	*Gobiodon erythrospilus*	Increase	Sørensen et al. (2014)
	*Oreochromis niloticus*	Increase	Fernandes and Rantin (1989)
	*Cyprinus carpio*	Increase	Ott *et al*. (1980)
	*Oncorhynchus mykiss*	Increase	Ott *et al*. (1980)
	*Pomacentrus moluccensis*	Increase	Nilsson *et al*. (2010)
	*Ostorhinchus doederleini*	Increase	Nilsson *et al*. (2010)
	*Carassius auratus grandoculis*	No effect	Yamanaka *et al*. (2013)
	*Etheostoma boschungi*	Decrease	Ultsch *et al*. (1978)
	*Etheostoma fusiforme*	Decrease	Ultsch *et al*. (1978)
	*Etheostoma flabellare*	Decrease	Ultsch *et al*. (1978)
	*Etheostoma rufilineatum*	Decrease	Ultsch *et al*. (1978)
Increasing salinity			
	*Cottus asper*	Decrease	Henriksson *et al*. (2008)
	*Leptocottus armatus*	No effect	Henriksson *et al*. (2008)
	*Cyprinus carpio*	Increase	De Boeck et al. (2000)
	*Cyprinodon ariegatus*	Increase	Haney and Nordlie (1997)
Increased $P_{CO_{2}}$			
	*Fundulus heteroclitus*	No effect	Cochran and Burnett (1996)
	*Leiostomus xanthurus*	No effect	Cochran and Burnett (1996)
	*Anguilla anguilla*	Increase	Cruz-Neto and Steffensen (1997)
	*Platichthys flesus*	Increase	Rogers (2015)
Hypoxic acclimation			
	*Pagrus auratus*	No effect	Cook *et al*. (2013)
	*S. salar*	No effect	Remen *et al*. (2013)
	*Hemiscyllium ocellatum*	Decrease	Routley *et al*. (2002)
	*Spinibarbus sinensis*	Decrease	Dan *et al*. (2014)
	*C. auratus*	Decrease	Fu *et al*. (2011)
	*Poecilia latipinna*	Decrease	Timmerman and Chapman (2004 a,b)
Reared in hypoxic environment			
	*Pseudocrenilabrus multicolor*	Decrease	Reardon and Chapman (2010)
Exercise pre-conditioning			
	*C. auratus*	Decrease	Fu *et al*. (2011)
Fed			
	*Astronotus ocellatus*	Increase	De Boeck et al. (2013)
	*Oreochromis niloticus*	Increase	Mamun *et al*. (2013)
	*Perca fluviatilis*	Increase	Thuy *et al*. (2010)
Fatty acid-enriched diet			
	*Solea solea* (larvae)	Decrease	McKenzie *et al*. (2008)
	*S. solea* (juveniles)	Decrease	McKenzie *et al*. (2008)
Increasing body mass			
	*Hypostomus plecostomus*	Decrease	Perna and Fernandes (1996)
	*Astronotus ocellatus*	Decrease	Sloman *et al*. (2006)
	*Pomacentridae*	No effect	Nilsson and Östlund-Nilsson (2008)
Pre- to post-settlement (larvae)			
	*Chromis atripectoralis*	Decrease	Nilsson *et al*. (2007a,b)
	*Pomacentrus amboinensis*	Decrease	Nilsson *et al*. (2007a,b)
Larvae to juveniles			
	*C. auratus grandoculis*	Decrease	Yamanaka *et al*. (2013)
Juveniles to adults			
	*Reinhardtius hippoglossoides*	Decrease	Dupont-Prinet *et al*. (2013)
Increasing brood size (mouthbrooders)			
	*Zoramia fragilis*	Increase	Östlun-Nilsson and Nilsson (2004)
	*Zoramia leptacantha*	Increase	Östlun-Nilsson and Nilsson (2004)
Mycobacteriosis infection			
	*Morone saxatilis*	Increase	Lapointe *et al*. (2014)
Acidified water			
	*Salmo gairdneri*	Increase	Ultsch *et al.* (1980)
	*Cyprinus carpio*	Increase	Ultsch *et al.* (1980)
Metal exposure			
	*Brycon amazonicus*	Increase	Monteiro *et al*. (2013) (Hg^2+^)
	*C. carassius*	Increase	Schjolden *et al*. (2007) (Cu^2+^)
	*Perca fluviatilis*	Increase	Bilberg *et al*. (2010) (AgNO_3_)
	*P. fluviatilis*	Increase	Bilberg *et al*. (2010) (nano-Ag)
Organophosphate exposure			
	*Oreochromis niloticus*	Increase	Thomaz *et al*. (2009)
Anaemia			
	*Pagrus auratus*	Increase	Cook *et al*. (2011)

Abbreviations: $P_{CO_{2}}$, partial pressure of carbon dioxide; *P*_crit_, critical oxygen level.

The stepwise multiple linear regression found that biotic (body mass, RMR) and abiotic (temperature, salinity) variables were highly correlated with *P*_crit_ (see Table 4). A significant regression (*F*_4,1154_ = 10.565, *P* < 0.001) predicted 19.5% of the variation in the data, based on an adjusted *r*^2^ (multiple linear regression). Predicted *P*_crit_ is equal to 5.689 + 0.047 (salinity) − 0.083(temperature) + 1.931(body mass) + 0.001 (RMR), where salinity is measured in practical salinity units, temperature in degrees Celsius, body mass in kilograms, and RMR in milligrams of oxygen per litre. All four variables were significant predictors of *P*_crit_ in the full model (Table 4).

**Table 4:** Results of the stepwise linear regression analysis where salinity, body mass, routine metabolic rate (RMR) and temperature had zero-order *r* correlations with *P*_crit_ (*P* < 0.05) and with each other, where values were reported

	Zero-order *r* (*n* = 159)							
Variable	Salinity (psu)	Temperature (°C)	Body mass (kg)	RMR (mg O_2_ l^−1^)	*P*_crit_ (kPa)	β	*sr^2^*	b
Salinity		0.317	−0.165	0.354	0.279	0.346	0.099	0.047
Temperature				0.366	−0.141	−0.314	0.081	−0.083
Body mass				−0.166	0.166	0.242	0.056	1.931
RMR					0.17	0.202	0.032	0.001
Mean	23.54	23.1	0.1	323.84	5.4	Intercept = 4.027		
SD	15.36	7.9	0.3	434.04	2.1	Adjusted *r*^2^ = 0.195	*P* < 0.001	

Abbreviations: *P*_crit_, critical oxygen level; RMR, routine metabolic rate. In the full model, all four variables were significant predictors of *P*_crit_.

**Figure 3:**
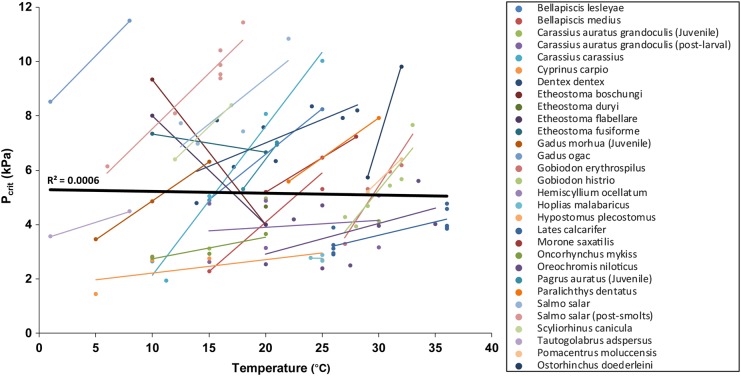
The effect of temperature on inter-species critical oxygen level (*P*_crit_; black dashed line) and intra-species *P*_crit_ (continuous lines).

Temperature is by far the most widely studied abiotic factor potentially interacting with hypoxia (reported in 30 species) and is particularly relevant, given ongoing global climate change (Ficke *et al*., 2007; Pörtner, 2010). As ectotherms, oxygen demand in fishes increases in a roughly exponential manner with temperature (inter-species mean *Q*_10_ of 1.83; Clarke and Johnston, 1999), and the intrinsic link between temperature and environmental hypoxia has become the basis of an overarching concept termed ‘oxygen and capacity limitation of thermal tolerance’ (Pörtner, 2001, 2010). Essentially, this concept suggests that the thermal tolerance of ectotherms is dictated by their capacity to meet the oxygen demands of aerobic metabolism. Increased temperature both elevates basal oxygen demand (SMR) and reduces oxygen supply (via its effect on oxygen solubility), whereas hypoxia reduces the oxygen supply. Hence, temperature and hypoxia are likely to act synergistically in fishes. Within species, increasing temperature generally results in a higher *P*_crit_, but among species, the slope of the relationship between temperature and *P*_crit_ is highly variable (Fig. 3). For example, the Atlantic salmon (*S. salar*) exhibits a steep linear increase of *P*_crit_ in comparison to the shallower slope seen in the common carp (*C. carpio*) across a similar temperature range (Ott *et al*., 1980; Remen *et al*., 2013). A surprising exception to the generally positive intra-species correlation between temperature and *P*_crit_ was observed in four out of six species of darter (*Etheostoma*), for which *P*_crit_ was lower at 20 than 10°C (Ultsch *et al*., 1978). Variation in the sensitivity of species to temperature in terms of hypoxia tolerance may arise because of differences in their potential for thermal acclimation. Explanations for this variation may include reducing the metabolic impact of increased temperature or enhancing oxygen extraction capacity (Ott *et al*., 1980; Pörtner, 2010). Species exhibit highly contrasting capacities for plastic acclimation responses. At opposite ends of this spectrum, crucian carp (*Carassius carassius*) can dramatically increase respiratory surface area through gill remodelling in response to temperature and hypoxia (Sollid *et al*., 2005), whereas certain tropical reef fish species (*Ostorhinchus doederleini* and *Pomacentrus moluccensis*) demonstrate no thermal acclimation ability even over a relatively modest temperature range (29–32°C; Nilsson *et al*., 2010).

Unlike intra-species *P*_crit_, there is no apparent relationship between temperature and inter-species *P*_crit_ (Fig. 3), suggesting that evolution may have nullified the thermal sensitivity of hypoxia tolerance across species. It has been shown that the difference in RMR between a typical cold-water and warm-water fish is less than expected, given the thermal sensitivity of RMR within individual species (intra-species median *Q*_10_ = 2.4; Clarke and Johnston, 1999). In addition, gill surface area appears to scale in a linear manner with metabolic rate, implying that natural selection equips fishes with the oxygen extraction capacity required to match demand at higher temperatures (Nilsson and Östlund-Nilsson, 2008). Selective pressures for small gills, such as the osmorespiratory compromise (Nilsson, 1986; Gonzalez and McDonald, 1992; Urbina and Glover, 2015), gill parasites and risks associated with gill injury, are likely to limit respiratory surface area so that oxygen extraction capacity does not exceed that required by a particular species for survival in its natural range (Nilsson, 2007). Thus, generalizations regarding hypoxia tolerance across temperatures cannot be established firmly at the inter-species level.

**Figure 4:**
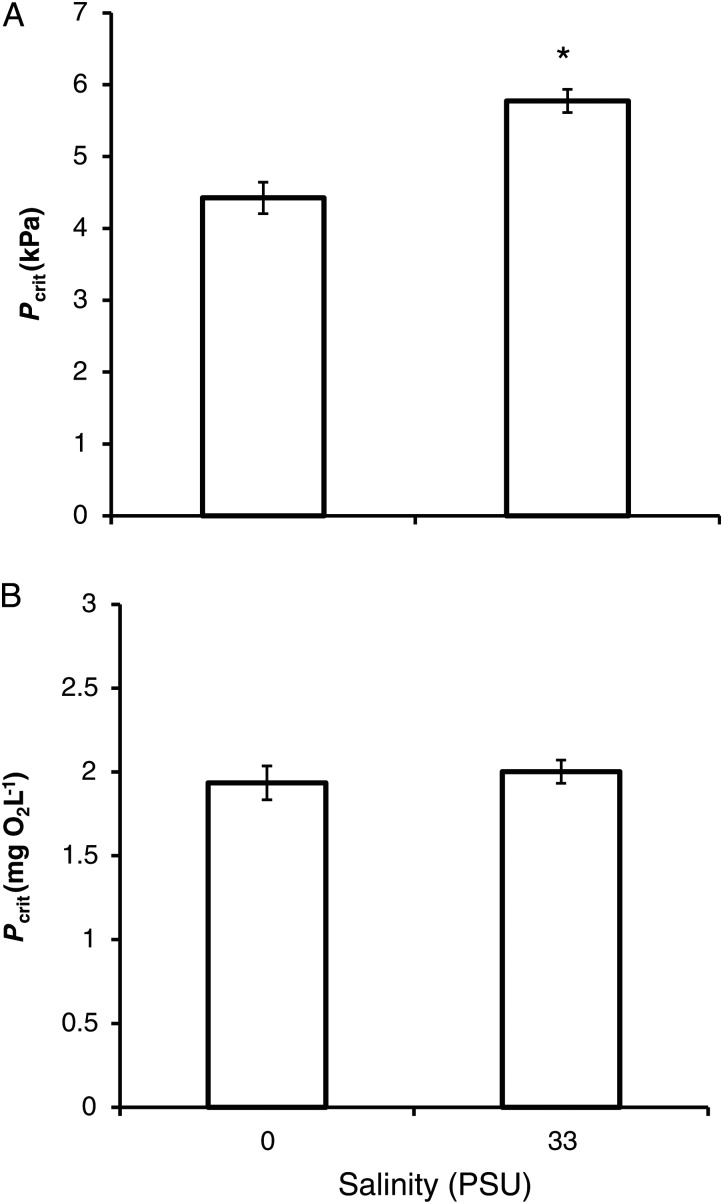
The effect of environmental salinity on inter-species critical oxygen level (*P*_crit_), expressed as partial pressure of oxygen (in kilopascals; **A**) and concentration of oxygen (in milligrams per litre; **B**). Data are shown as means + SEM, including data from 82 species in seawater and 50 species in freshwater. *Unpaired *t*-test, significant when *P* < 0.050.

Although salinity has long been recognized as a key environmental factor, studies evaluating the effects of salinity on *P*_crit_ are scarce. A previous study in the euryhaline sheephead minnow (*Ciprinodon variegatus*), acclimated to salinities from freshwater (0 PSU) to hypersaline waters (100 PSU), showed a marked effect on *P*_crit_ (Haney and Nordlie 1997) as environmental salinity rose. Inter-specific comparisons in the database agree with this previous intra-specific finding; that is, salinity had a significant influence on *P*_crit_, whereby freshwater species (including a few euryhaline species) presented a 23% lower *P*_crit_ than seawater species (also including a few euryhaline species; Fig. 4A; *P* ≤ 0.001).

As explained in earlier sections, any factor influencing the oxygen demand (metabolic rate) of an individual will be likely to have implications for its hypoxia tolerance. Given that teleost fishes must maintain a tight regulation of their internal salts and water composition (osmolality), as external salinity changes or becomes extreme, fishes must expend increased efforts to maintain internal homeostasis (Urbina and Glover, 2015). As many of the mechanisms of osmoregulation involve the action of ATP-driven pumps (i.e. Na^+^,K^+^-ATPase) in order to pump ions against a concentration gradient, increased costs of osmoregulation may explain, in part, some of these differences in *P*_crit_, at least for intra-specific comparisons. However, from our database (inter-specific), where more freshwater vs. seawater species comparison are presented, it is likely that other mechanisms are explaining differences in *P*_crit_. Given that seawater species separated million years ago from a freshwater ancestor (actinoptyergians, 300–180 million years ago; Vega and Wiens, 2012), both fresh- and seawater species have adapted to their respective environments, and therefore, have also optimized their energy allocated to osmoregulation. Thus, the differences in *P*_crit_ found in the present study, rather than being explained by energy-related/oxygen demand issues, could be associated with intrinsic characteristics of both media (freshwater vs. seawater). Owing to differences in size, organic matter load and stability, hypoxia is much more prevalent and common in freshwater than in seawater environments. As such, the driver for an enhanced hypoxia tolerance (lower *P*_crit_) could potentially explain the lower *P*_crit_ found in freshwater species. A future phylogenetic analysis might contribute to test this hypothesis.

It is also worth noting that the difference found in *P*_crit_ when presented as the partial pressure of oxygen (in kilopascals) was no longer found when *P*_crit_ was calculated as the concentration (in milligrams per litre; (Fig. 4B; *P* > 0.05). This could potentially highlight the importance of working with partial pressure, because this is what drives diffusion when considering gases. Alternatively, it could indicate that the oxygen concentration is more relevant when considering *P*_crit_ values, because it determines the total amount of oxygen that is potentially available for diffusion as water flows over the gills, i.e. for the same oxygen uptake, salinity (through its effect on solubility) will have a big effect on the difference between inspired and expired $P_{O_{2}}$.

The biological processes that consume O_2_ also produce CO_2_; therefore, hypoxia and hypercarbia can often co-occur in aquatic environments (Ultsch, 1996; Cruz-Neto and Steffensen, 1997; Gilmour, 2001). Despite this, the interactive effect of environmental hypercarbia on hypoxia tolerance has been relatively understudied. As previously discussed (Table 3), there are conflicting reports within the available literature regarding to the effect of hypercarbia on the *P*_crit_ of fishes (Cochran and Burnett, 1996; Cruz-Neto and Steffensen, 1997; McKenzie *et al*., 2003). The most likely mechanism by which hypercarbia could negatively impact hypoxia tolerance is through respiratory acidosis, leading to Bohr/Root effects on haemoglobin and reduced oxygen transport capacity (Jensen *et al*., 1993; Cruz-Neto and Steffensen, 1997). In this respect, hypercarbia is partly akin to the far more extreme acidosis that can occur in poorly buffered freshwater environments subjected to acid precipitation or drainage. Acidification of the surrounding water by addition of sulphuric acid (water pH range 7.4–4.0, at constant atmospheric $P_{CO_{2}}$) increases *P*_crit_ in both rainbow trout (*Oncorhynchus mykiss*) and common carp (*Cyprinus carpio*; Ultsch *et al*., 1980). The time required to compensate for acid–base disturbance is highly variable among species (10–24 h during moderate hypercarbia; Melzner *et al*., 2009), and as such, the effect of hypercarbia and acidification on hypoxia tolerance is likely to be dependent largely on the species in question as well as the severity and duration of the hypercarbic or acid exposure (Jensen *et al*., 1993).

Exposure to toxicants, such as trace metal contamination, appears to reduce hypoxia tolerance in fishes. Specifically, exposure to elevated concentrations of copper (300 µg l^−1^), mercury (150 µg l^−1^) and silver (63 µg l^−1^) have been demonstrated to increase *P*_crit_ in various species (Table 3). The accumulation of toxic metals on the gills can stimulate the hypersecretion of mucus, which acts as a barrier to diffusion of external toxicants into the blood (McDonald and Wood, 1993; Wilson *et al*., 1994). In addition, some trace metals cause hyperplasia and hypertrophy of gill epithelia cells that results in the fusing and thickening of gill lamellae (Schjolden *et al*., 2007; Bilberg *et al*., 2010). As a consequence, respiratory function is compromised as a result of reduced diffusion area and increased diffusion distance (McDonald and Wood, 1993). The organophosphate insecticide trichlorfon has been shown to increase *P*_crit_ by inducing similar changes in gill morphology as well as by promoting vasoconstriction that reduces lamellar blood flow in Nile tilapia (*Oreochromis niloticus*; Thomaz *et al*., 2009). These potential interactions between toxic contaminants and hypoxia in fishes are clearly of concern, particularly given that both stressors predominantly threaten freshwater and coastal marine systems and are therefore likely to coincide (McDonald and Wood, 1993; Diaz and Rosenberg, 2008).

Determinations of *P*_crit_ in fishes have almost universally been made in unfed, post-absorptive individuals which, although providing a useful basis for comparing absolute hypoxia tolerance among species and individuals, does not fully account for the digestive state typical of fishes in their natural setting. An increase in oxygen uptake following ingestion of food, termed specific dynamic action (SDA), is required in order to meet the energetic costs associated with mechanical and biochemical digestion and assimilation (Jobling, 1993). Shortly after a meal, oxygen uptake in fish typically rises rapidly, reaching a peak two to three times higher than pre-fed levels within a few hours. The shape and duration of the SDA is highly dependent on the species in question as well as the meal size and composition (Secor, 2009). Measurements of *P*_crit_ in fishes undergoing SDA have revealed significant increases in *P*_crit_ compared with unfed control fishes, showing that increased aerobic demand during digestion has negative consequences for hypoxia tolerance (Table 3). In common perch (*Perca fluviatilis*) force-fed a 5% body mass ration, *P*_crit_ at 20 h post-feeding was increased by 1.44-fold compared with sham-fed individuals (Thuy *et al*., 2010). Likewise, oscars (*Astronotus ocellatus*) fasted for 14 days showed a 1.6-fold lower *P*_crit_ than individuals fed a daily 1% body mass ration up to 24 h prior to *P*_crit_ determination (De Boeck et al., 2013). In such experiments, the requirement for a stable $M_{O_{2}}$ on which to base a determination of *P*_crit_ means that measurements at peak SDA are not feasible, and thus, are likely to underestimate the effect of digestion on hypoxia tolerance (Thuy *et al*., 2010).

Several studies have investigated the effect of hypoxia acclimation on *P*_crit_ (Table3). Broadly, short-term physiological acclimation to hypoxia appears to be achieved through either enhanced O_2_ extraction capacity or metabolic depression. In goldfish (*Carassius auratus*), 48 h of severe (0.63 kPa) hypoxia induced dramatic increases in both lamellar surface area and blood haemoglobin content, leading to a 49% reduction in *P*_crit_ compared with individuals held at normoxia (Fu *et al*., 2011). Likewise, sailfin molly (*Poecilia latipinna*) demonstrated increased haemoglobin and red blood cell concentrations and a reduced *P*_crit_ following a 6 week exposure to severe hypoxia (Timmerman and Chapman, 2004a). Depression of RMR at normoxia and a subsequent reduction in *P*_crit_ following chronic hypoxic exposure has been observed in the epaulette shark (*H. ocellatum*; Routley *et al*., 2002) and qingbo (*Spinibarbus sinensis*; Dan *et al*., 2014). However, some less hypoxia-tolerant species appear to demonstrate no physiological acclimation potential through hypoxic pre-conditioning. Daily exposure to 6 h of moderate hypoxia (10.5 kPa) for 33 days had no effect on *P*_crit_ in post-smolt Atlantic salmon (*S. salar*; Remen *et al*., 2013). Additionally, chronic (6 week) moderate hypoxia produced no change in the *P*_crit_ of juvenile snapper (*Pagrus auratus*; Cook *et al*., 2013).

As hypoxia is likely to become an increasingly predominant aquatic perturbation in the future (Vaquer-Sunyer and Duarte, 2008; Keeling *et al*., 2009), the degree of physiological plasticity for hypoxia tolerance will be a key determinant of species performance. The potential for long-term and transgenerational hypoxia acclimation with respect to *P*_crit_ has been largely unstudied. A transgenerational transfer of hypoxia tolerance has been demonstrated in zebrafish (*Danio rerio*) larvae after 2–4 weeks of parental hypoxia exposure, but this was based on determinations of time to loss of equilibrium (4 kPa O_2_) rather than through measurement of *P*_crit_ (Ho and Burggren, 2012). Reardon and Chapman (2010) demonstrated a strong element of developmental plasticity in the *P*_crit_ of the Egyptian mouthbrooder (*Pseudocrenilabrus multicolor*) when reared in hypoxic conditions. In addition, intra-species population effects on *P*_crit_ across habitats of differing O_2_ regimens have been observed in several species, indicating that a high degree of phenotypic plasticity for *P*_crit_ exists within these populations (Timmerman and Chapman, 2004b; Reardon and Chapman 2010; Fu *et al*., 2011).

### Future applications

The comprehensive *P*_crit_ database presented here provides the opportunity for a variety of further analyses with potential to offer fundamental physiological, as well as wider ecological, insights. For example, further analyses could involve comparing species *P*_crit_ values within a phylogenetic context as a means to investigate the evolutionary relationships of hypoxia tolerance among species (Mandic *et al*., 2009). Likewise, combining species *P*_crit_ data with information on the spatial distribution of populations would help to refine our understanding of the ecological relevance of *P*_crit_ as a physiological trait. Such an analysis would be particularly relevant to predicting the impacts on fish populations likely to arise from the increasingly widespread occurrence of hypoxic zones in aquatic environments around the globe (Friedrich *et al*., 2014). Given the variability found in the reported *P*_crit_ for different fish species, it is likely that hypoxic events will have consequences that are very dependent on individual species. This highlights the complexity of predicting the effects that hypoxia will have at community and ecosystem levels, and the potential for hypoxia to have differential effects on predator-prey interactions, migrations, and ultimately, global fisheries.

The integration of the present database with similar databases of other widely measured physiological parameters in fishes should offer useful insights into interactions among traits. Such physiological data are of great value for improving the predictive capacity of models as an aid to the management and conservation of aquatic systems (Jørgensen et al., 2012; Cooke *et al*., 2013). Traits for which databases are currently under construction include the metabolic response to feeding (SDA), aerobic scope, growth rate and critical temperature. On completion, the combined data set will be made widely accessible via an online data repository facility, such as that provided by Dryad (http://datadryad.org/). Thus, it is envisaged that these data will prove to be a tangible link between the field of fish physiology and future studies of ecology, conservation and management.

## Supplementary material

Supplementary material is available at *Conservation Physiology* online.

## Funding

This work was supported by a Natural Environment Research Council (NERC, UK) PhD studentship awarded to N.J.R./R.W.W. and NERC and Biotechnology and Biological Sciences Research Council (BBSRC) research grants (NE/H010041/1, BB/D005108/1 and BB/J00913X/1) awarded to R.W.W. The physiological database is a contribution of the European Union Cooperation in Science and Technology (COST) Action (FA1004) on the ‘Conservation Physiology of Marine Fishes’. The same EU COST Action supported this work as a Short Term Scientific Mission (STSM). For more information, see: http://fish-conservation.nu/.

## Supplementary Material

Supplementary Data
