# Supplementary material for: A new analysis of hypoxia tolerance in fishes using a database of critical oxygen level (Pcrit)
Source: Conserv Physiol. 2016 Apr 27;4(1):cow012. doi: 10.1093/conphys/cow012 (PMC4849809; doi:10.1093/conphys/cow012)
Supplement: Supplementary Data [file cow012supp.zip › cow012supp.docx]

**Supplementary Data**


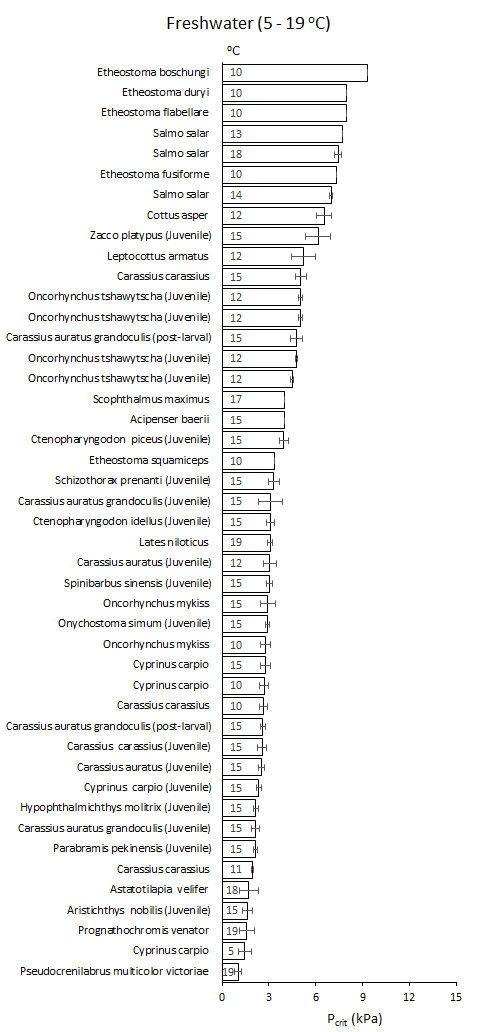


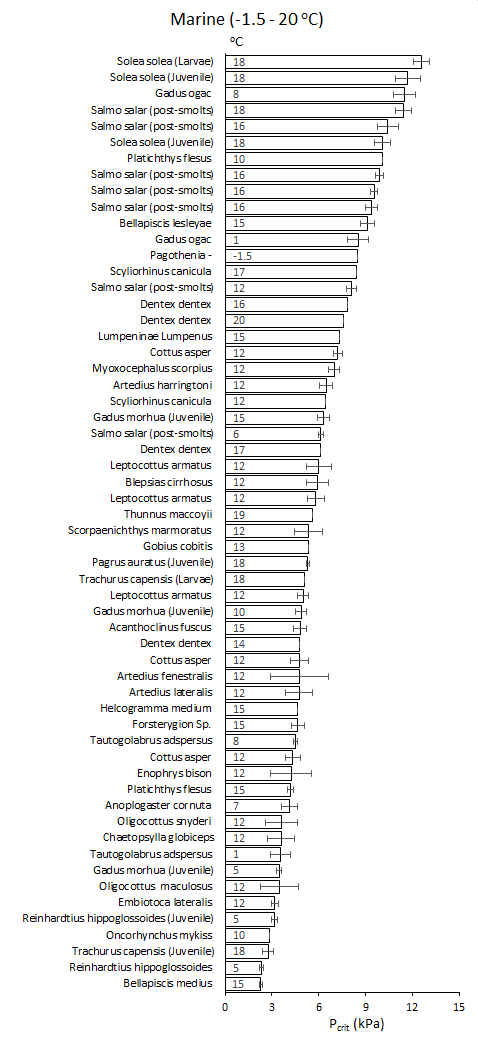


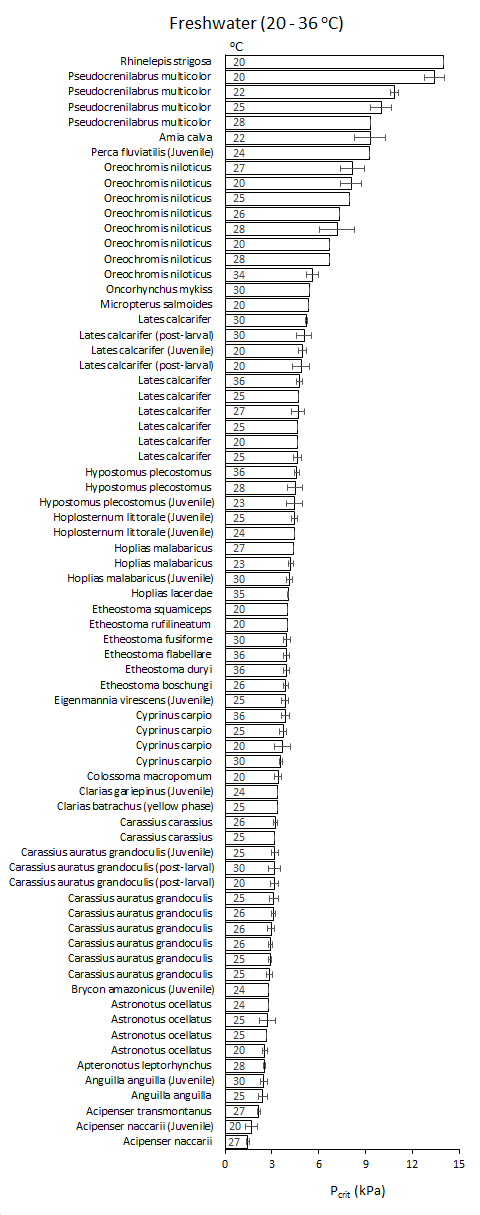


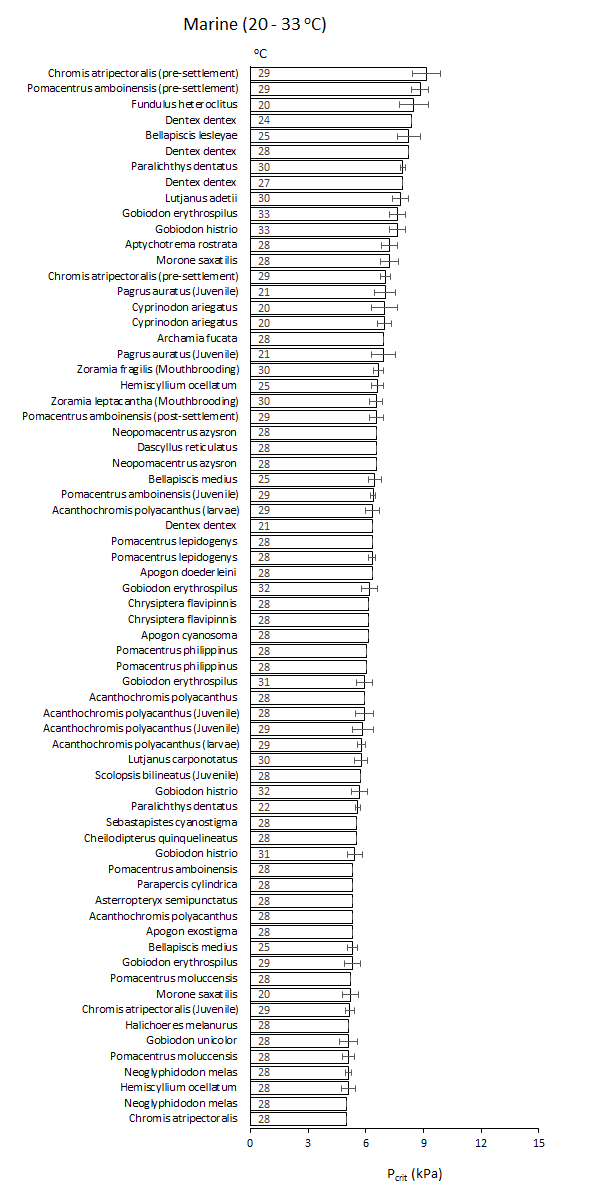


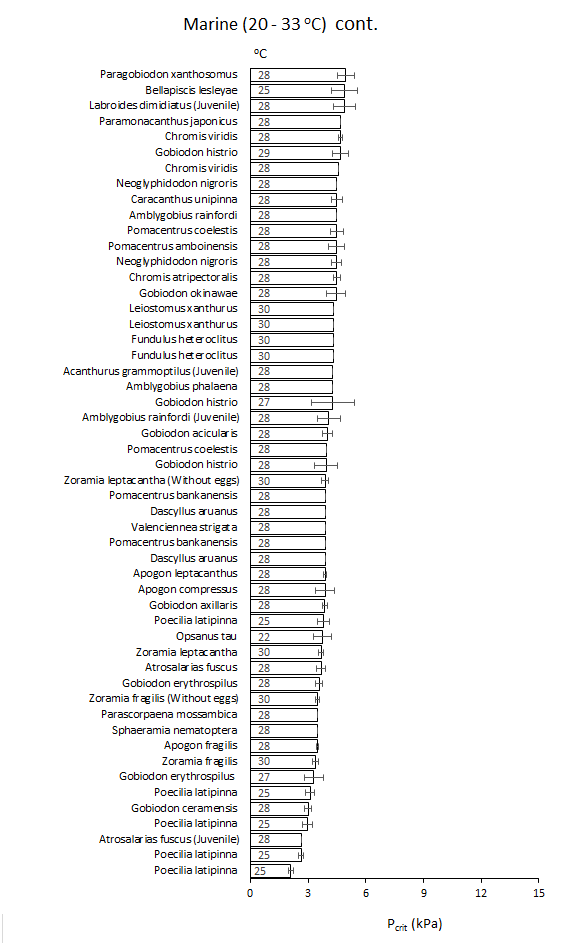


Supplementary Figure 1. Plots of the species and their respective mean *P*_crit_ (± SE) values that were incorporated into the ‘control’ dataset. Numbers contained within each bar indicate the temperature (°C) at which *P*_crit_ was determined. Data are ordered by *P*_crit_ (highest to lowest) and grouped by temperature range and water type (marine / freshwater).
